# Immunomodulatory Effect of Vitamin C on Proinflammatory Cytokines Production in Ossimi Lambs (*Ovis aries*) with Pneumonic Pasteurellosis

**DOI:** 10.3390/ani11123374

**Published:** 2021-11-25

**Authors:** Mohamed Abdo Rizk, Shimaa Abd El-Salam El-Sayed, Doaa Salman, Basma H. Marghani, Hossam Elshahat Gadalla, Mohamed Z. Sayed-Ahmed

**Affiliations:** 1 Department of Internal Medicine and Infectious Diseases, Faculty of Veterinary Medicine, Mansoura University, Mansoura 35516, Egypt; drzakaria-infect@hotmail.com; 2 Department of Biochemistry and Chemistry of Nutrition, Faculty of Veterinary Medicine, Mansoura University, Mansoura 35516, Egypt; shimaa_a@mans.edu.eg; 3 Department of Animal Medicine, Faculty of Veterinary Medicine, Sohag University, Sohag 82524, Egypt; abassdoaa@yahoo.com; 4 Department of Physiology, Faculty of Veterinary Medicine, Mansoura University, Mansoura 35516, Egypt; basmahamed@mans.edu.eg; 5 Department of Clinical Pathology, Faculty of Veterinary Medicine, Mansoura University, Mansoura 35516, Egypt; gadallaha@mans.edu.eg; 6 Department of Clinical Pharmacy, College of Pharmacy, Jazan University, Jizan 82722, Saudi Arabia

**Keywords:** vitamin C, lambs, pneumonic pasteurellosis, cytokines, Egypt

## Abstract

**Simple Summary:**

There is paucity on the immunomodulatory and anti-inflammatory effects of vitamin C on cytokines production in lambs with respiratory diseases. Therefore, the current study examined the influence of vitamin C on the production of different pro-inflammatory cytokines in lambs naturally infected by pneumonic pasteurellosis. In lambs naturally infected with pneumonic pasteurellosis, the association between measured proinflammatory cytokines and clinical sum score demonstrated a positive correlation with interleukin-6 and interferon gamma. The findings show that vitamin C has a selective effect on the production of pro-inflammatory cytokines in sera of lambs with pneumonic pasteurellosis when used in combination with tulathromycin, and confirming the therapeutic effectiveness of tulathromycin in clinical recovery of lambs with pneumonic pasteurellosis.

**Abstract:**

In this study, we have investigated the impact of vitamin C on the production of pro-inflammatory cytokines (interleukin 1 β (IL-1 β), interleukin 6 (IL-6), interleukin 12p40 (IL-12p40), interferon gamma (IFNγ), and tumor necrosis factor alpha (TNF-α)) in lambs naturally infected by pneumonic pasteurellosis. Of 37 lambs, 18 lambs were identified to have pneumonic pasteurellosis and randomly allocated into two equal groups. Single subcutaneous dose of tulathromycine alone (2.5 mg kg^−1^) or tulathromycine combined with vitamin C (3 gm kg^−1^) were administrated to the diseased lambs. The serum levels of IL-1β, IL-6, IFN-γ, and TNF-α were returned to the normal levels in pneumonic lambs treated with the combination therapy. The obtained results indicate the selective influences of vitamin C on pro-inflammatory cytokines production in sera of lambs with pneumonic pasteurellosis and highlights the value of vitamin C as a potential anti-inflammatory drug and ideal immunomodulatory agent.

## 1. Introduction

Pneumonia is an inflammation of the pulmonary alveoli in response to infective agents and causes great economic losses in ovine industry worldwide [1]. Many bacterial and viral infectious agents are incriminated in disease occurrence in lambs. Of note, *Pasteurella* species are common in sheep and cause two main diseases; Pneumonic pasteurellosis and systemic pasteurellosis [2]. The multifactorial dynamic nature of pneumonia gives the chance to these opportunistic pathogens that inhabit the upper respiratory tract of animals to become pathogenic when the animal is exposed to either stressful environment or infection with primary respiratory pathogens, such as viral agents or *Mycoplasma* spp. [1,3]. Tumor necrosis factor alpha (TNF-α), interleukin-1 beta (IL-1β), and interleukin-8 (IL-8) begin and regulate interaction between cytokines, leukocytes, vascular endothelium, cellular adhesion molecules, and soluble chemotactic factors in inflamed tissue [4,5]. Microbial pathogens and other stimuli act as inducer to the secretion of TNF-α and IL-1β from monocytes and macrophages [4]. As a result, in the treatment of clinical cases, administering immunostimulant drugs to diseased animals while researching the response of animal immunity to such compounds is a top priority. Vitamin C (ascorbic acid) is a water-soluble nutrient found in the extracellular fluid and the cytosolic compartment of the cell that primarily affects host defence mechanisms and immunological homeostasis in this regard [6]. Immunomodulation is aimed primarily to improve the host resistance against microbes or other infectious agents by overcoming the immunosuppressive effects of stress [7]. Immunomodulatory agents can exert such action through neutralization of the pro-inflammatory cytokines (IL-2, IFN-γ, IL-12, tumor necrosis factor (TNF)-α, IL-1β, and IL-17) [8]. Previous studies reported the significant role of micronutrients in immunomodulation and their impact on treatment of immunodeficiency diseases [7,9,10]. Interestingly, vitamin C is one of the most critical micronutrients that is used as an immunostimulant immunomodulatory agent [7]. At the same time, there is scarcity of information on the relationship between sera cytokines and naturally occurring pneumonic pasteurellosis in lambs. Therefore, this study describes for the first time the effect of vitamin C as an immunomodulatory agent on the pro-inflammatory cytokines production in sera samples of healthy lambs and in those infected by pneumonic pasteurellosis. In response to infection, vitamin C’s potential immunostimulant impact induces increased T-lymphocyte proliferation and inhibits T cell death signalling pathways [11,12,13]. In vitro, the screened L-ascorbic acid inhibited bovine *Pasteurella multocida* A growth and virulence expression, and supplementation significantly increased the survival rate of mice and reduced the bacterial load in vivo, implying that L-ascorbic acid could be used as a potential protective agent for bovine *Pasteurella multocida* A infection in the clinic [14]. Furthermore, vitamin C’s effectiveness as a prophylactic and therapeutic agent in the treatment of scurvy, viral infections, and the common cold [7], and cancer [15], and atherosclerosis [16] were reported. However, there is extremely limited information on the effects of vitamin C on the production of cytokines in lambs with respiratory diseases. Therefore, in this study we have investigated the impact of vitamin C on the production of pro-inflammatory cytokines (IL-1β, IL-6, IL-12p40, IFN-γ, and TNF-α) in lambs naturally infected by pneumonic pasteurellosis. To the best of the authors’ knowledge, this is the first study to explore the effect of vitamin C on cytokines levels in lambs with pneumonic pasteurellosis under natural field conditions.

## 2. Materials and Methods

### 2.1. Animals and Clinical Examination

Forty-seven Ossimi lambs (*Ovis aries*) (37 diseased and 10 healthy) belonging to a free-grazing sheep flock (*n* = 251) in El-Dakahlia province, Egypt, were used. The average age and body weight of animals under study were 65 ± 10 days old and 19 ± 4 kg, respectively. Lambs with pneumonic pasteurellosis were selected according to medical history, direct observation, clinical signs (recurrent fever, depression with congested mucous membrane, serous and seromucoid nasal discharge, frequent moist cough, anorexia, polypnea, tachycardia, and abnormal lung sound), physical examination, nasopharyngeal swabs, and bronchoalveolar lavages [3,5,17]. Clinical sum scoring for all signs (CIIS) was performed as previously described by Love et al. [18] as fellows; cough 0 = none; 1 = single induced, 2 = multiple induced, and 3 = multiple spontaneous; nasal discharge 0 = none; 1 = small amount of unilateral cloudy discharge, 2 = bilateral, cloudy, or excessive mucus discharge, and 3 = copious bilateral mucopurulent discharge; ocular discharge 0 = none; 1 = small amount of ocular discharge, 2 = moderate amount of bilateral discharge, and 3 = heavy ocular discharge; rectal temperature (°C) 0 = ≤40.5; 1 = 40.6–41.5, 2 = 41.6–42.5, and 3 = ≥42.6; breath rate (breath/min) 0 ≤ 70, 1 = 70.1–80, 2 = 80.1–90, and 3 ≥ 90.1; heart rate (beat/min) 0 ≤ 160, 1= 160.1–170, 2 = 170.1–180, and 3 ≥ 180.

### 2.2. Blood Sampling and Bacteriological Investigations

Venous blood samples were collected via jugular venipuncture from each animal after disinfecting the puncture area with ethyl alcohol 70% into a Vacutainer tube without anticoagulant for sera separation from healthy lambs (*n* = 10) and those identified with pneumonic pasteurellosis (*n* = 18). Next, the separated sera samples were stored at −20 °C until biochemical analyses. For isolation and identification of *Pasteurella multocida* (*P. multocida*)*,* samples were collected from all animals (*n* = 47) and inoculated onto 7% sheep blood agar and MacConkey agar plates. Then the plates were incubated aerobically at 37 °C for 24–48 h following the standard bacteriological techniques previously described by Carter and Cole [19]. After that, standard microbiological techniques [19] were used for isolates identification. Diseased animals were recognised as lambs with clinical signs of pneumonia and a positive culture for *P. multocida*. The lambs identified with pneumonic pasteurellosis (*n* = 18) were tested for the presence of respiratory viral infection as bovine virus diarrhea (BVD) as previously detailed [20,21]. Reverse transcription polymerase chain reaction (RT-PCR) assay was used to detect the presence or absence of BVD virus as previously detailed by Drew et al. [21]. Healthy controls were lambs with no clinical symptoms and negative culture results.

### 2.3. Cytokines Measurement in Blood Serum

Serum samples were used for estimation of IL-1β, IL-6, IL-12P40, TNF-α, and IFNγ (MyBiosource, Inc., San Diego, CA, USA) using commercially available sheep ELISA Kits according to the manufacturers’ recommendations. Samples were run in duplicate for all of the examined cytokines. Briefly, 100 µL of specific capture antibody for each cytokine was diluted in coating buffer and incubated overnight at 4 °C in microplate. Then, the plates were blocked with freshly prepared 10% fetal bovine serum (FBS) and incubated at room temperature (RT) for 1 h. Next, 100 µL of each standard, sample and control were prepared in the assay diluent and added to each well. Thereafter, the plates were sealed and incubated for 2 h at RT. Afterwards, 100 µL of detection antibody was added to each micro-well and incubated for 1 h at RT. Next, 100 μL a Horseradish Peroxidase (HRP) Conjugate was diluted into the assay diluent and added to each well and incubated for 30 min at 37 °C. After that, each well was filled with substrate reagent (Tetramethylbenzidine (TMB) and hydrogen peroxide) and incubated at room temperature for 30 min in the dark. Finally, after adding 50 µL of stopping reagent (1 M H3PO4), the reading was taken. Following each procedure, newly prepared wash buffer was used to aspirate and wash the contents of each well (phosphate buffers saline with 0.05 per cent Tween-20). On each immunoplate, the plates were read at 450 nm with a 570 nm correction wavelength. To calculate the amount of cytokine produced, a standard cytokine curve was run (Supplementary Material Figures S1–S5). Finally, calculated values were expressed in picograms per millilitre (pg/mL).

### 2.4. Structural Similarity Measurements

For calculating the structural similarity between tulathromycin, and vitamin c, atom Pair fingerprints (APfp) were used [22]. The APfp of the selected compounds was calculated using the CID obtained from the PubChem for each compound. The CIDs were there loaded into the ChemMine tools software for calculating APfp of all compounds [23].

### 2.5. Treatment Protocol

Diseased lambs (*n* = 18) were divided into two groups at random. The first group received single dose of antibiotic therapy tulathromycine (Draxxin^®^; Pfizer, Parsipanny, NJ, USA) at a dose rate of 2.5 mg kg^−1^ subcutaneous (S/C). Lambs in the second group were treated with a combination therapy consisting of 3 gm kg^−1^ vitamin C (Cevarol^®^; Memphis, Cairo, Egypt) and 2.5 mg kg^−1^ tulathromycine. Both drugs were administrated S/C in the same inoculation period.

### 2.6. Statistical Analysis

A statistical software application was used to analyse the data (JMP for windows Version 5.1; SAS Institute, Cary, NC, USA). For each analysed variable, mean values and standard deviation were computed. MANOVA repeated measures on treatment and time were used to evaluate the main effect of drug and time in the evaluation of treatment results. Wilks’ lambda test was chosen to assess within-group interactions as well as evidence of time-group interactions. When the Wilks’ lambda test revealed a statistically significant difference between groups, a one-way ANOVA with Tukey-Kramer HSD post-hoc multiple comparison testing was employed to determine whether group was statistically distinct from the others. The relationship between the total sums of clinical scores in lambs suffering from pneumonic pasteurellosis and proinflammatory cytokines was investigated using the Pearson correlation test coefficient. At *p* < 0.05, differences between means were judged significant.

## 3. Results

### 3.1. Vitamin C Effect on Clinical Findings

A total of 37 lambs exhibited respiratory manifestation in the current study. Bacteriological examination confirmed the infection by *P. multocida, M. haemolytica, E. coli* and *Staphylococcus* spp. in 18, 11, 6, and 2 lambs, respectively. RT-PCR result revealed that the selected 18 lambs were free from BVD (Figure S6).

**Figure 1:**
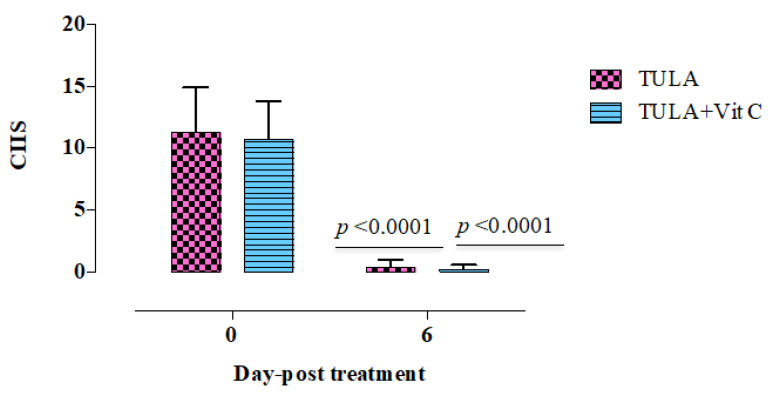
CIIS: the total sum of clinical scores in lambs suffering from pneumonic pasteurellosis and those treated with tulathromycin alone (*n* = 9), or combination of tulathromycin with vitamin C (*n* = 9). *p* < 0.0001; indicates statistical significant difference between treated groups before treatment and six days post treatment. Each value represents (mean ± SD). TULA, tulathromycine alone. TULA + vitamin C, combination therapy from tulathromycin and vitamin C.

The clinical appearance was assessed in the lambs identified by pneumonic pasteurellosis six days after treatment. Compared to healthy controls, the infected lambs had a substantial increase (*p* < 0.05) in body temperature, respiratory rate, and heart rate. Additionally, the diseased lambs exhibited abnormal lung sound and cough (Table 1). Interestingly, the mean body temperature, breath rate, and heart rate were returned to the normal levels in all treated groups (Figure 1). Treatment with the antibacterial therapy alone or in combination with vitamin C was associated with a significantly faster (*p* < 0.0001) improvement in CIIS (cough, nasal discharge, ocular discharge, breath rate, heart rate, and rectal temperature), on the sixth day of treatment (Figure 1). The disappearance of clinical symptoms on treated lambs on the sixth day post treatment confirmed the highly efficacy of tulathromycin/vitamin C combination in the treatment of respiratory disease in lambs.

### 3.2. Vitamin C Effect on Pro-Inflammatory Cytokines Levels in Sera

In comparison to the healthy control group, all assessed pro-inflammatory cytokines (IL-1, IL-6, IL-12p40, IFN-, and TNF-) in all diseased lambs showed significant increases (*p* < 0.05) (Table 2 and Table 3). Significant suppression (*p* < 0.05, Wilks’ lambda test for drug × time interaction, *p* < 0.0001) in the serum levels of all assessed pro-inflammatory cytokines was found in all treated lambs at six days post-treatment (Table 2 and Table 3). In pneumonic lambs treated with a combination therapy of tulathromycin and vitamin C, blood levels of IL-1, IL-6, IFN-, and TNF- were significantly reduced (*p* < 0.05) and restored to normal levels (Table 2 and Table 3). On contrary, no such reduction was observed in IL-12p40 serum levels treated lambs (Table 2). Interestingly, all measured pro-inflammatory cytokines were statistically significantly reduced (*p* < 0.05) in lambs received treatment by combination therapy than lambs treated by tulathromycin alone at six days post treatment (Table 2 and Table 3). The results confirmed the anti-inflammatory effect of tulathromycin and highlight the role of vitamin C in potentiating such effect in treated lambs.

### 3.3. Correlation between the Clinical Signs Scores and the Production of Pro-Inflammatory Cytokines

The correlation between assessed proinflammatory cytokines and CIIS in lambs naturally infected by pneumonic pasteurellosis revealed a positive correlation between CIIS and IL-6 and IFNγ (Table 4 and Table S1), while other estimated proinflammatory cytokines exhibited a negative correlation with CIIS (Table 4 and Table S1). The correlation between all the assessed proinflammatory cytokines in diseased lambs revealed appositive correlation between either IL-1 β and both IL-12p40 and TNF-α, IL-6, and IL-12p40, or TNF-α and IFNγ (Table 4 and Table S1). Lambs treated by tulathromycin revealed a positive correlation between CIIS and all assessed proinflammatory cytokines except TNF-α with a significant statistical correlation (*p* < 0.05) with IL-6 (Table 5 and Table S2). Treatment of lambs with tulathromycin and vitamin C combination therapy exhibited postive correlation between CIIS and IL-12p40, TNF-α, and IFNγ (Table 6 and Table S3).

## 4. Discussion

In Egypt, sheep has a major economic impact on livestock productivity. However, during winter season respiratory diseases are common among lambs and causes great economic losses [3,5].

Pro-inflammatory cytokines play a great synergistic role in the regulation of inflammatory process. Such synergism is applied by the synergistic production of TNF-α, IFN-γ, IL-1, IL-6, and IL-8 [24]. Significant elevation in pro-inflammatory cytokines including IL-1β, Il-6, IL-12p40, IFN-γ, and TNF-α were observed in this study. Similar findings were observed in the sera levels of IL1-β, TNF-α, and IFN-γ in pigs with viral respiratory disease [25] and in cattle with bacterial infection [26,27]. Moreover, elevated expression of IL1-β and TNF-α were detected in the respiratory airways and lung lesions of calves with pneumonic pasteurellosis [28]. Generally, the exaggerated production of such cytokines is controlled by several pathways, including the production of anti-inflammatory cytokines [29], cortisol [30], or administration of immunomodulatory agent that has an anti-inflammatory effect [7].

**Figure 2:**
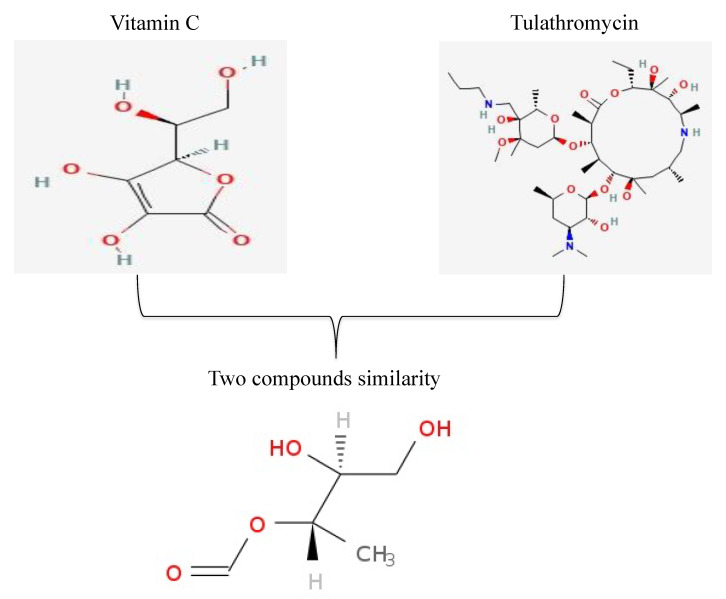
Tulathromycin with vitamin C structural similarities analysis. The chemical structures were obtained from the website (http://pubchem.ncbi.nlm.nih.gov/summary/summary.cgi?cid=2853195, accessed on 31 October 2021).

Basically, the repeated measures multivariate MANOVA is used to see if there are any variations in multiple dependent variables across time or between treatments, assuming that the variables have been measured at all time points and have participated in all treatments. Our results exhibited the significant role (MANOVA fit, *p* < 0.0001. Wilks’ lambda test for drug × time interaction, *p* < 0.0001) of vitamin C, a major water-soluble vitamin in normalization the pro-inflammatory cytokines (IL-1β, Il-6, IFN-γ, and TNF-α) when used in combination with tulathromycin in lambs with pneumonic pasteurellosis. Such findings revealed the immunomodulatory effect of vitamin C in such disease condition, which come in accordance with previous studies that approved this effect against *Pasteurella monodon* [31] and *Rachycentron canadum* [32]. Taken together, the compatible property of vitamin C with commonly used anti-microbial agent (tulathromycin), the well-known chemical composition and biological activity of vitamin C, the non-toxic dose of vitamin C even at high dose for animals and humans, and its inexpensive cost, we can conclude that vitamin C is an ideal immunomodulatory agent for lambs naturally infected by *P. multocida*. Similar to vitamin C, honey and its related products have the capacity to regulate the secretion of cytokines from monocytes and macrophages and regulate the generation of ROS from neutrophils [33]. Therefore, the current study opens the gate to administrate such product in combination with low doses of antimicrobials in treatment of veterinary infectious diseases. Structural similarity measurements between the selected antimicrobial agent, tulathromycin, and vitamin C revealed fingerprint bits 0.02 with 0.15 maximum common substructure (MCS) (Figure 2).

Tebbe et al. [34] confirmed the inhibitory effect of vitamin C on the release of IL-1α and IL-6 in keratinocytes. Our results revealed the potential of vitamin C and tulathromycin combination to normalize all selected pro-inflammatory cytokines except IL-12p40. Such discrepancy may indicate the selective role of vitamin C on pro-inflammatory cytokines inhibitions. Therefore, further studies are required to elucidate why pro-inflammatory cytokines are selectively influenced by vitamin C in lambs with pneumonic pasteurellosis. Understanding the vitamin C impact on pro-inflammatory cytokine production in such cases is prospective to generate novel insight into the pathology of airways inflammation and could provide several insinuations for the clinical use of vitamin C as a potential anti-inflammatory drug under field conditions.

NF-κB (nuclear factor kappa-light-chain-enhancer of activated B cells) is a protein complex present in all animal cell and controls cytokine production in response to stimuli such as stress, cytokines, and bacterial or viral antigens [35]. Surprising, TNF- alpha and IL-6 had been reported to activate the production of NF-κB [35]. Taken together, the inhibitory effect of vitamin C on TNF- alpha and IL-6 in this study, we can suggest that vitamin C has a role in inhibition the autocrine stimulation pathways of pro-inflammatory cytokines via NFkB.

Study limitations should be mentioned. Further studies are required to approve the role of vitamin C in inhibition the autocrine stimulation pathways of pro-inflammatory cytokines via NFkB either in vitro or in experimentally infected lambs. Although this study investigated the immunomodulatory effect of vitamin C on proinflammatory cytokines production in lambs with pneumonic pasteurellosis, the study examined the selected lambs for the presence of BVD virus with neglection of other viruses causing respiratory manifestations in lambs as parainfluenza-3 or mycoplasma infection as *Mycoplasma capricolum subspecies capripneumoniae, Mycoplasma ovipneumoniae,* and *Mycoplasma arginine*. This study did not investigate whether the anti-inflammatory and immunomodulating effects of vitamin C on pneumonic lambs when used in combination with tulathromycin are direct or indirect, or whether such a selective inhibitory effect of vitamin C on pro-inflammatory cytokine production is limited to tulathromycin alone or in combination with other antimicrobial agents. As a result, more research is needed to investigate this discrepancy and to examine the probable synergistic effect of the medications employed when administered together. This analysis will aid in determining the most efficient component ratio for inhibiting *P. mutlocida* growth in lambs for clinical use.

## 5. Conclusions

The obtained results indicate the selective influences of vitamin C when used in combination with tulathromycin on the production pro-inflammatory cytokines in sera of lambs with pneumonic pasteurellosis and highlights the value of such combination as a potential anti-inflammatory therapy with confirmation the therapeutic effectiveness of tulathromycin in clinical recovery of lambs with respiratory diseases. Moreover, the obtained results revealed the anti-inflammatory and immunomodulating effects of vita min C on pneumonic lambs when used in combination with tulathromycin, with high light ing the possible synergistic action between tulathromycin and vitamin C.

## Figures and Tables

**Table 1:** Clinical findings associated with pneumonic pasteurellosis in 18 Ossimi lambs in comparison with 10 healthy lambs.

Variables	Healthy Lambs (*n* = 10)	Diseases Lambs (*n* = 18)
Respiratory rate (breath/min)	53.27 ± 3.01	91.05 ± 4.73 *
Heart rate (beat/min)	125.11 ± 1.48	166.23 ± 8.19 *
Rectal temperature (°C)	39.05 ± 1.02	42.17 ± 0.82 *
Chest sound	Normal breath sound (*n* = 10)	Abnormal breath sound (exaggerated vesicular (*n* = 3), wheezes (*n* = 4), crackles (*n* = 3), mixed (*n* = 8)
Cough	Absent (*n* = 10)	Present (*n* = 18)

* *p* < 0.05.

**Table 2:** Interleukins value in Ossimi lambs treated with tulathromycin alone (GP I) or tulathromycin combined with vitamin C (GP II).

	IL-1 β		IL-6		IL-12p40	
	Time Post-Treatment (Day)					
	Zero	6	Zero	6	Zero	6
Healthy lambs (*n* = 10)	36.49 (21.06–78.84) ^b^	42.36 (27.21–80.13) ^b^	53.95 (26.51–68.35) ^b^	47.64 (23.66–78.55) ^b^	39.10 (33–45.74) ^b^	38.605 (30.24–48.42) ^c^
GP I (*n* = 9)	270.22 (250.376–204.21) ^a^	130.47 (107.30–204.21) ^a^	685.55 (514.915–831.6) ^a^	294.49 (203.83–385.17) ^a^	369.47 (229.17–418.21) ^a^	220.24 (210–257.11) ^a^
GP II (*n* = 9)	271 (248–308) ^a^	35.76 (31.33–128.39) ^b^	582.07 (411.79–846.205) ^a^	59.48 (34.875–88.69) ^b^	319.68 (239.36–410.11) ^a^	197.22 (127–204.64) ^b^

Variables with different superscript letters in the same column are significantly different at *p* < 0.05. The obtained values represent the median and range in each group in each treated group. MANOVA fit, *p* < 0.0001. Wilks’ lambda test for drug × time interaction, *p* < 0.0001. Abbreviations: interleukin 1 β (IL-1 β), interleukin 6 (IL-6), and interleukin 12p40 (IL-12p40).

**Table 3:** Interferon gamma (IFNγ) and tumor necrosis factor alpha (TNF-α) values in Ossimi lambs treated with tulathromycin alone (GP I) or tulathromycin combined with vitamin C (GP II).

	IFNγ		TNF-α	
	Time Post-Treatment (Day)			
	Zero	6	Zero	6
Healthy lambs (*n* = 10)	45.22 (32.07–58.21) ^b^	42.63 (30.33–55.43) ^b^	17.45 (13.11–42.89) ^b^	21.95 (2–48.01) ^b^
GP I (*n* = 9)	417.12 (263. 88–627.56) ^a^	107.368 (101.009–205.472) ^a^	587.27 (503.37–713.11) ^a^	213.21 (133.45–377.53) ^a^
GP II (*n* = 9)	436.52 (298.98–645. 68) ^a^	65 (50.684–91.37) ^b^	603.37 (481.06–749.22) ^a^	73.8 (41.93–91.33) ^b^

Variables with different superscript letters in the same column are significantly different at *p* < 0.05. The obtained values represent the median and range in each group in each treated group. MANOVA fit, *p* < 0.0001. Wilks’ lambda test for drug × time interaction, *p* < 0.0001.

**Table 4:** Correlation between clinical sum scores and the production of pro-inflammatory cytokines in lambs naturally infected with pneumonic pasteurellosis.

Variables	CIIS	IL-1 β	IL-6	IL-12p40	TNF-α	IFNγ
CIIS	1.00	−0.14	0.10	−0.23	−0.19	0.06
IL-1 β	−0.14	1.00	−0.23	0.20	0.05	−0.28
IL-6	0.10	−0.23	1.00	0.38	−0.06	−0.02
IL-12p40	−0.23	0.20	0.38	1.00	−0.40	−0.64
TNF-α	−0.19	0.05	−0.06	−0.40	1.00	0.41
IFNγ	0.06	−0.28	−0.02	−0.64	0.41	1.00

The final obtained data are the *r* value using Pearson correlation test coefficient. Abbreviations: CIIS (clinical sum scores), interleukin 1 β (IL-1 β), interleukin 6 (IL-6), interleukin 12p40 (IL-12p40), interferon gamma (IFNγ), and tumor necrosis factor alpha (TNF-α). Differences between means at *p* < 0.05 were considered significant.

**Table 5:** Correlation between the clinical sum scores and the production of pro-inflammatory cytokines in lambs treated with tulathromycin.

Variables	CIIS	IL-1 β	IL-6	IL-12p40	TNF-α	IFNγ
CIIS	1.00	0.43	0.80	0.65	−0.31	0.19
IL-1 β	0.43	1.00	0.63	0.18	−0.55	0.70
IL-6	0.80	0.63	1.00	0.61	−0.57	0.23
IL-12p40	0.65	0.18	0.61	1.00	−0.44	−0.04
TNF-α	−0.31	−0.55	−0.57	−0.44	1.00	−0.25
IFNγ	0.19	0.70	0.23	−0.04	−0.25	1.00

The final obtained data are the *r* value using Pearson correlation test coefficient. Abbreviations: CIIS (clinical sum scores), interleukin 1 β (IL-1 β), interleukin 6 (IL-6), interleukin 12p40 (IL-12p40), interferon gamma (IFNγ), and tumor necrosis factor alpha (TNF-α). Differences between means at *p* < 0.05 were considered significant.

**Table 6:** Correlation between the clinical sum scores and the production of pro-inflammatory cytokines in lambs treated with tulathromycin combined with vitamin C.

Variables	CIIS	IL-1 β	IL-6	IL-12p40	TNF-α	IFNγ
CIIS	1.00	−0.23	−0.33	0.03	0.42	0.29
IL-1 β	−0.23	1.00	−0.05	−0.03	−0.34	0.37
IL-6	−0.33	−0.05	1.00	−0.36	−0.49	−0.20
IL-12p40	0.03	−0.03	−0.36	1.00	−0.28	0.02
TNF-α	0.42	−0.34	−0.49	−0.28	1.00	−0.33
IFNγ	0.29	0.37	−0.20	0.02	−0.33	1.00

The final obtained data are the *r* value using Pearson correlation test coefficient. Abbreviations: CIIS (clinical sum scores), interleukin 1 β (IL-1 β), interleukin 6 (IL-6), interleukin 12p40 (IL-12p40), interferon gamma (IFNγ), and tumor necrosis factor alpha (TNF-α). Differences between means at *p* < 0.05 were considered significant.

## Data Availability

On reasonable request, the corresponding author will provide the datasets created and/or analyzed during the current work.

## Supplementary Materials

The following are available online at https://www.mdpi.com/article/10.3390/ani11123374/s1. Figure S1. Standard cytokine curve for interleukin 1 β (IL-1 β), Figure S2. Standard cytokine curve for interleukin 6 (IL-6), Figure S3. Standard cytokine curve for interleukin 12p40 (IL-12p40), Figure S4. Standard cytokine curve for interferon gamma (IFNγ), Figure S5. Standard cytokine curve for tumor necrosis factor alpha (TNF-α), Figure S6. RT-PCR product visualized after agarose gel electrophoresis. The expected size of the PCR products was 290 bp. PC, Positive control sample, NC, Negative control, M = 100 bp ladder. RT-PCR was used to confirm the infection by BVD in the pooled buffy coat samples collected from the selected lambs. Cytopathic strain of BVDV genotype-2 (Strain 125) were obtained from the department of Rinderpest like Diseases, Veterinary Serum and Vaccine Research Institute, Cairo and used as a control strain for RT-PCR test. Pure RNA was extracted using viral RNA Purification Kit following the manufacturer protocol. PCR cycling conditions were performed using the following PCR primer sets: the forward primer (5′-ATGCCCTTAGTAGGACTAGCA-3′)/reverse primer (5′- CAACTCCATGTGCCATGTACAGCAG -3′). Table S1. P values for the Pearson correlation test coefficient between clinical signs scores and the production of pro-inflammatory cytokines in lambs naturally infected with pneumonic pasteurellosis, Table S2. P values for the Pearson correlation test coefficient between responses to the treatment by tulathromycin in lambs naturally infected with pneumonic pasteurellosis, Table S3. P values for the Pearson correlation test coefficient between responses to the treatment by tulathromycin combined with vitamin C in lambs naturally infected with pneumonic pasteurellosis
