# Supplementary material for: Immunomodulatory Effect of Vitamin C on Proinflammatory Cytokines Production in Ossimi Lambs (Ovis aries) with Pneumonic Pasteurellosis
Source: Animals (Basel). 2021 Nov 25;11(12):3374. doi: 10.3390/ani11123374 (PMC8697947; doi:10.3390/ani11123374)
Supplement: Supplementary file 1 [file animals-11-03374-s001.zip › animals-1373634-supplementary.pdf]

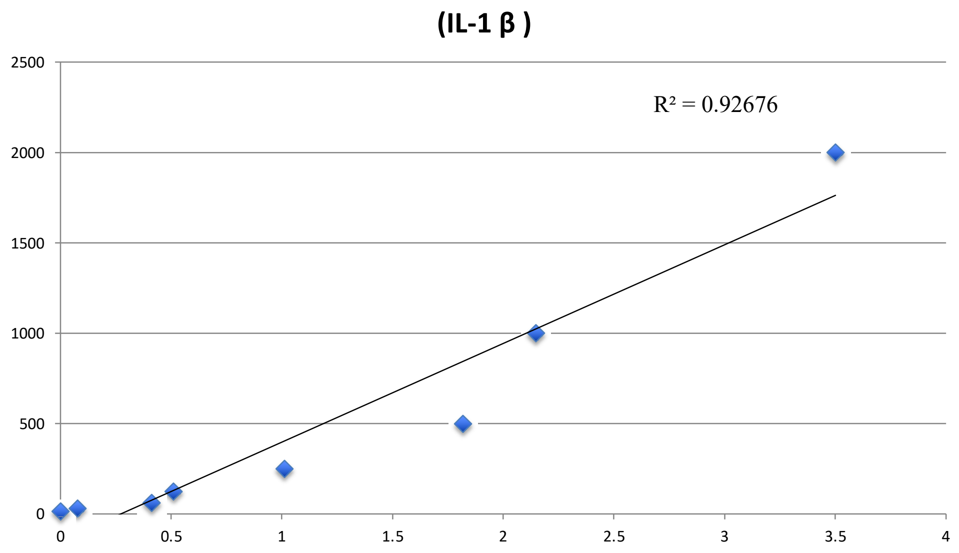

Figure S1. Standard cytokine curve for interleukin 1 β (IL-1 β)

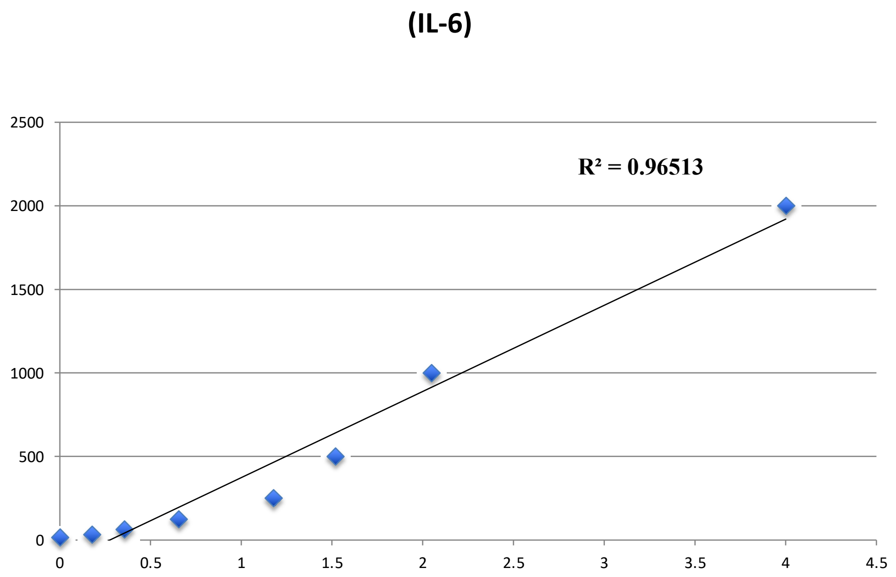

Figure S2. Standard cytokine curve for interleukin 6 (IL-6)

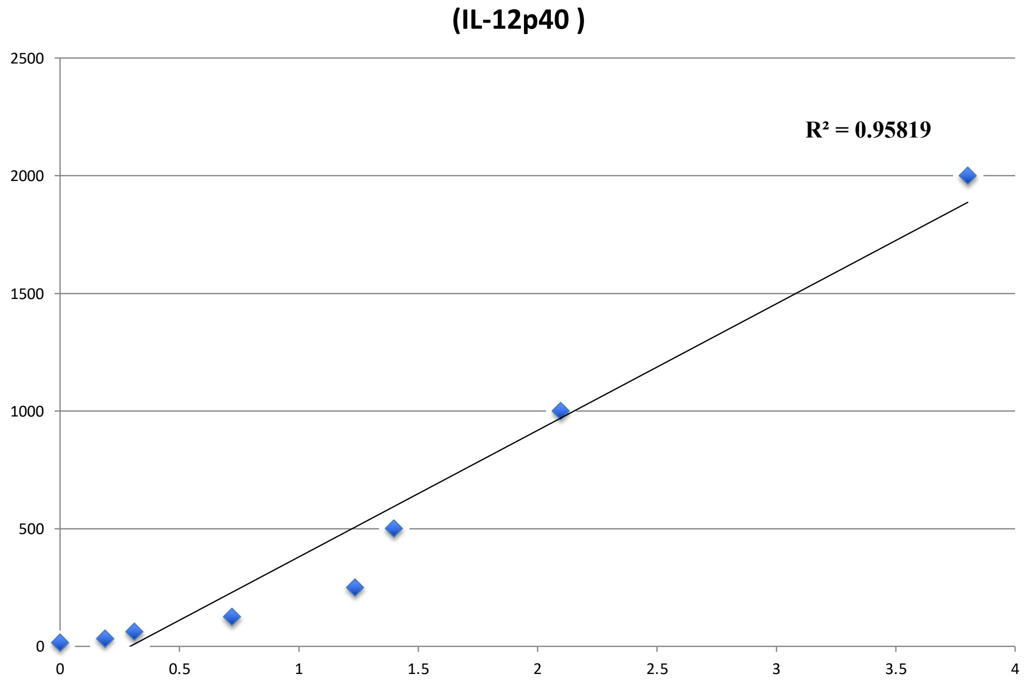

Figure S3. Standard cytokine curve for interleukin 12p40 (IL-12p40)

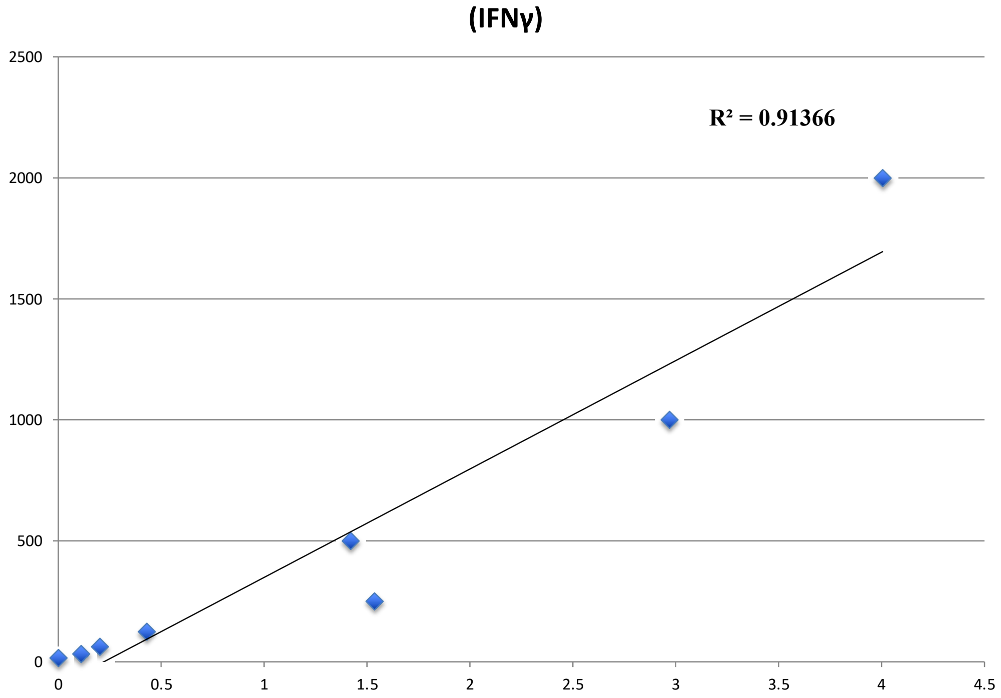

Figure S4. Standard cytokine curve for interferon gamma (IFNγ)

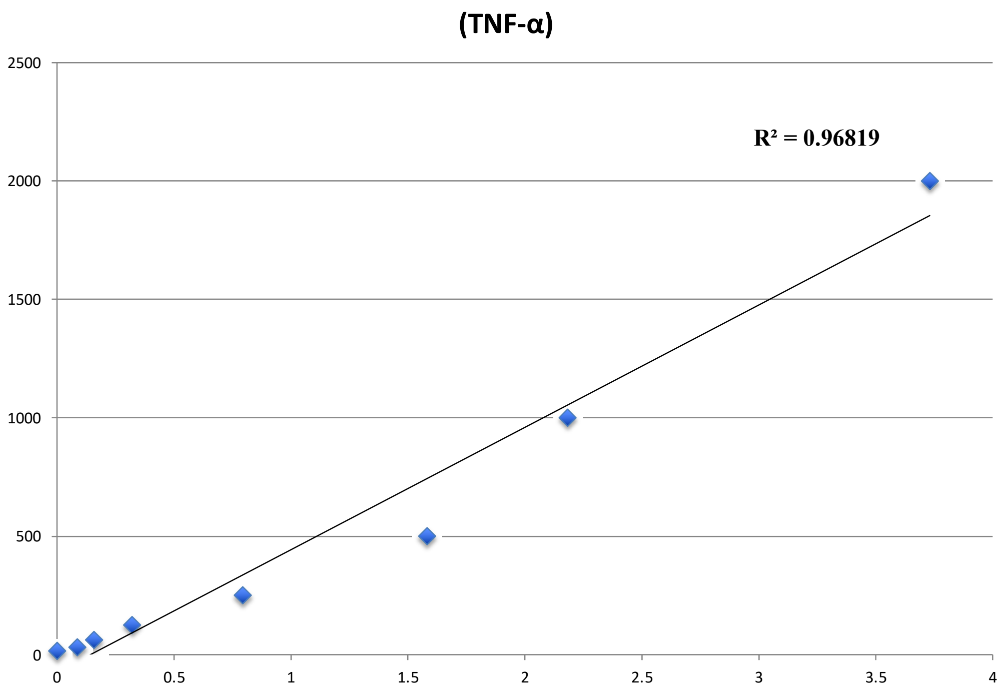

Figure S5. Standard cytokine curve for tumor necrosis factor alpha (TNF-α)

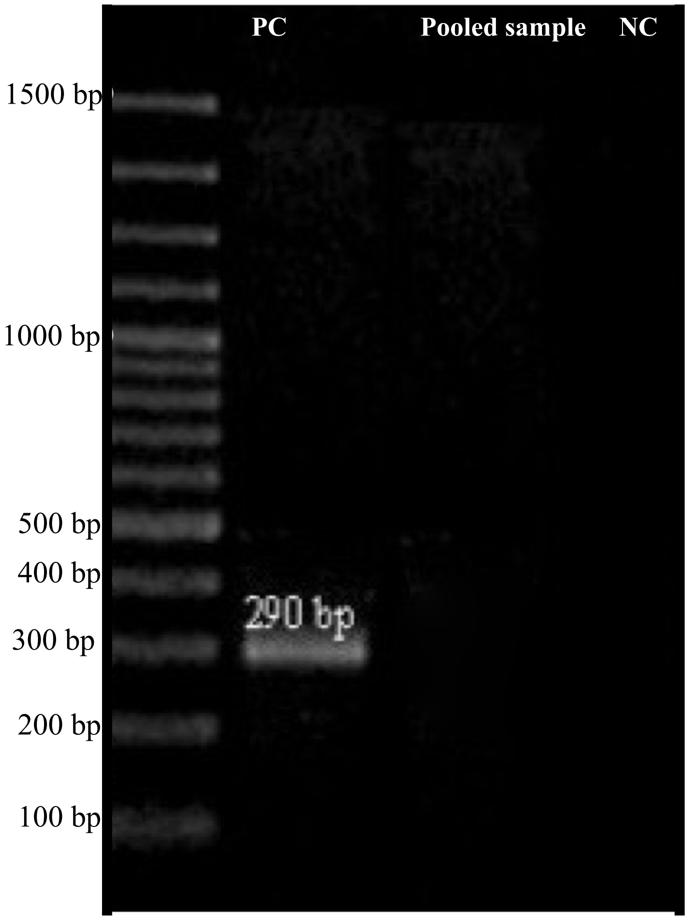

Figure S6. RT-PCR product visualized after agarose gel electrophoresis. The expected size of the PCR products was 290 bp. PC, Positive control sample, NC, Negative control, M= 100 bp ladder. RT-PCR was used to confirm the infection by BVD in the pooled buffy coat samples collected from the selected lambs. Cytopathic strain of BVDV genotype-2 (Strain 125) were obtained from the department of Rinderpest like Diseases, Veterinary Serum and Vaccine Research Institute, Cairo and used as a control strain for RT-PCR test. Pure RNA was extracted using viral RNA Purification Kit following the manufacturer protocol. PCR cycling conditions were performed using the following PCR primer sets: the forward primer (5′-ATGCCCTTAGTAGGACTAGCA-3′)/reverse primer (5′- CAACTCCATGTGCCATGTACAGCAG -3′).

Table S1. *P* values for the Pearson correlation test coefficient between clinical signs scores and the production of pro-inflammatory cytokines in lambs naturally infected with pneumonic pasteurellosis

**Variables**	**CSS**	**IL-1 β**	**IL-6**	**IL-12p40**	**TNF-α**	**IFNγ**
**CSS**		0.57	0.70	0.36	0.44	0.80
**IL-1 β**	0.57		0.35	0.42	0.84	0.26
**IL-6**	0.70	0.35		0.12	0.81	0.93

**IL-12p40**	0.36	0.42	0.12		0.09	**0.004**
**TNF-α**	0.44	0.84	0.81	0.09		0.09
**IFNγ**	0.80	0.26	0.93	**0.004**	0.09	

Abbreviations: CSS (clinical signs score), interleukin 1 β (IL-1 β), interleukin 6 (IL-6), interleukin 12p40 (IL-12p40), interferon gamma (IFNγ) and tumor necrosis factor alpha (TNF-α). Differences between means at $P < 0.05$ were considered significant.

Table S2. *P* values for the Pearson correlation test coefficient between responses to the treatment by tulathromycin in lambs naturally infected with pneumonic pasteurellosis

**Variables**	**CSS**	**IL-1 β**	**IL-6**	**IL-12p40**	**TNF-α**	**IFNγ**
**CSS**		0.25	**0.009**	0.05	0.41	0.63
**IL-1 β**	0.25		0.06	0.64	0.13	**0.03**
**IL-6**	**0.009**	0.06		0.08	0.11	0.55
**IL-12p40**	0.05	0.64	0.08		0.23	0.93
**TNF-α**	0.41	0.13	0.11	0.23		0.52
**IFNγ**	0.63	**0.03**	0.55	0.92	0.52	

Abbreviations: CSS (clinical signs score), interleukin 1 β (IL-1 β), interleukin 6 (IL-6), interleukin 12p40 (IL-12p40), interferon gamma (IFNγ) and tumor necrosis factor alpha (TNF-α). Differences between means at $P < 0.05$ were considered significant.

Table S3. *P* values for the Pearson correlation test coefficient between responses to the treatment by tulathromycin combined with vitamin C in lambs naturally infected with pneumonic pasteurellosis

**Variables**	**CSS**	**IL-1 β**	**IL-6**	**IL-12p40**	**TNF-α**	**IFNγ**
**CSS**		0.55	0.38	0.93	0.25	0.44

**IL-1 β**	0.55		0.90	0.94	0.37	0.32
**IL-6**	0.38	0.90		0.34	0.17	0.61
**IL-12p40**	0.93	0.94	0.34		0.46	0.95
**TNF-α**	0.25	0.37	0.17	0.46		0.38
**IFNγ**	0.44	0.32	0.61	0.95	0.38	

Abbreviations: CSS (clinical signs score), interleukin 1 β (IL-1 β), interleukin 6 (IL-6), interleukin 12p40 (IL-12p40), interferon gamma (IFNγ) and tumor necrosis factor alpha (TNF-α). Differences between means at $P < 0.05$ were considered significant.
